# Poisoned by Vegetables from Poisoned Soils

**Published:** 1880-12

**Authors:** W. R. Munroe

**Affiliations:** Baltimore, Md.


ARTICLE IV.

### POISONED BY VEGETABLES FROM POISONED SOILS.

BY W. R. MUNROE, M. D., BALTIMORE, MD.

As it has been published in several newspaper periodicals during the year that individuals had been poisoned and several had died from eating certain vegetables, such as potatoes and melons which had been grown in soils where Paris Green had been used too freely for the purpose of killing certain bugs which infested the vines; it would be interesting and instructive to be informed by chemical analysis, or otherwise, how far or to what extent the soil of the ground is capable of eliminating from and imparting to vegetable productions poisonous substances deposited into the earth, either to destroy vermin or to fertilize the ground.

In one of our public institutions, about a year since, it was discovered that a bloody flux was prevailing extensively among its inmates, which prompted the managers of said institution to employ the services of a Chemist to ferret out, if possible, the cause of that terrible malady. In his analysis, that chemist was successful in discovering raw and unchanged night soil deposited in the cells of the vegetables grown in the garden of that Institution, and of which the inmates had freely eaten, which had produced the bloody flux of which so many suffered. The night soil had been put in that garden in its natural and crude state, and in large quantities. The ground could not digest it.

As a sanitary precaution not only to public institutions, but to all citizens who purchase vegetables in our public markets, ought not this to be thoroughly investigated? It may be that some gardeners, through ignorance, use too freely unmanipulated night soils, or other substances which are deleterious to the production of wholesome vegetables, and the maintenance of good health. Will you suggest any precaution that may be taken to prevent the growth and sale of poisonous vegetables?

[NOTE.—We would be pleased to publish answers to Dr. Munroe's inquiries from some of our readers who have had occasion to examine into the subject. It certainly is a matter of grave importance, and is yearly becoming more so to the inhabitants of our cities, and large towns, who are compelled to depend to a great extent upon the truck farms in their vicinity, for their vegetable supplies.]—*Ed. Ind. Prac.*

